# Dominant Cone-Rod Dystrophy: A Mouse Model Generated by Gene Targeting of the GCAP1/*Guca1a* Gene

**DOI:** 10.1371/journal.pone.0018089

**Published:** 2011-03-28

**Authors:** Prateek K. Buch, Marija Mihelec, Phillippa Cottrill, Susan E. Wilkie, Rachael A. Pearson, Yanai Duran, Emma L. West, Michel Michaelides, Robin R. Ali, David M. Hunt

**Affiliations:** 1 University College London Institute of Ophthalmology, London, United Kingdom; 2 School of Animal Biology, University of Western Australia, Perth, Western Australia, Australia; Pennsylvania State University, United States of America

## Abstract

Cone dystrophy 3 (COD3) is a severe dominantly inherited retinal degeneration caused by missense mutations in *GUCA1A*, the gene encoding Guanylate Cyclase Activating Protein 1 (GCAP1). The role of GCAP1 in controlling cyclic nucleotide levels in photoreceptors has largely been elucidated using knock-out mice, but the disease pathology in these mice cannot be extrapolated directly to COD3 as this involves altered, rather than loss of, GCAP1 function. Therefore, in order to evaluate the pathology of this dominant disorder, we have introduced a point mutation into the murine *Guca1a* gene that causes an E155G amino acid substitution; this is one of the disease-causing mutations found in COD3 patients. Disease progression in this novel mouse model of cone dystrophy was determined by a variety of techniques including electroretinography (ERG), retinal histology, immunohistochemistry and measurement of cGMP levels. It was established that although retinal development was normal up to 3 months of age, there was a subsequent progressive decline in retinal function, with a far greater alteration in cone than rod responses, associated with a corresponding loss of photoreceptors. In addition, we have demonstrated that accumulation of cyclic GMP precedes the observed retinal degeneration and is likely to contribute to the disease mechanism. Importantly, this knock-in mutant mouse has many features in common with the human disease, thereby making it an excellent model to further probe disease pathogenesis and investigate therapeutic interventions.

## Introduction

Inherited cone dystrophies affect around 1/10,000 people [1]. Patients typically present with progressive loss of central vision and reduced colour vision in the second to third decades of life [2]. Mutations in several genes are now known to cause inherited cone dystrophies [2]. The first such mutation to be identified was in *GUCA1A* encoding Guanylate Cyclase Activating Protein 1 (GCAP1), a component of the phototransduction cascade [3]. GCAP1 is expressed in both rod and cone photoreceptors, where it plays a critical role in recovery following photon capture, by stimulating retinal Guanylate Cyclase (retGC1) to produce cyclic GMP (cGMP) in a Ca^2+^-dependent manner [4]–[6]. GCAP1 acts as a Ca^2+^ sensor by binding Ca^2+^ ions to three of its four EF-hand domains when the intracellular Ca^2+^ concentration rises to around 500 nM in the ‘dark state’; in its Ca^2+^-bound state GCAP1 represses retGC1 activity, ensuring that cGMP levels do not rise above the 10–30 µM needed to keep the cGMP-gated (CNG) cation channels open [6]–[8]. Following the light-activated hydrolysis of cGMP, the CNG cation channels close thereby reducing influx of Ca^2+^. Since Ca^2+^ continues to be extruded, this results in a reduction in intracellular Ca^2+^ concentration and the dissociation of Ca^2+^ ions from GCAP1, which in its unbound state permits retGC-mediated cGMP production. This increase in cGMP results in the opening of the CNG cation channels, allowing the influx of Ca^2+^ ions and a restoration of the resting levels of cyclase activity.

The first mutation in *GUCA1A* to be associated with dominant cone dystrophy was an A-to-G missense mutation in codon 99 that results in the replacement of tyrosine with cysteine (Y99C) in EF3 of the GCAP1 protein [3]. The binding of Ca^2+^ ions to GCAP1 is thereby reduced, making the cyclase activity less responsive to Ca^2+^ concentration and leading to constitutive activation of retGC1 by the mutant GCAP1 [9]. Subsequently, several other mutations in *GUCA1A* have been shown to alter amino acids predicted to form or stabilise Ca^2+^-binding EF-hand motifs in GCAP1, thereby causing a similar loss of Ca^2+^-sensitivity and constitutive activation of retGC-mediated cGMP production (reviewed in [10]). All have been associated with dominant cone dystrophy [11]–[15].

Although these mutations in *GUCA1A* affect the function of GCAP1 protein by a common mechanism, there is considerable inter- and intra-familial heterogeneity in the associated clinical phenotypes. In the majority of cases, patients carrying mutations in *GUCA1A* have little or no reduction in peripheral visual field and night vision, but experience marked photophobia and significantly impaired colour vision and visual acuity – consistent with the sparing of rod function and the classification of disease as a cone dystrophy. However, a number of patients with mutations in *GUCA1A* have reduced rod as well as cone function; whilst the disorder in most patients with Y99C and L151F mutations is reported as cone dystrophy with normal rod-mediated vision, some affected individuals from the same families have altered rod function and present as a cone-rod dystrophy [14], [16]. It is unclear why mutations that result in similar biochemical defects have such varied clinical sequelae; specifically, it is not known whether rod involvement in some patients is a direct result of the mutations in *GUCA1A*, or due to the presence of modifier loci.

Animal models of disease have provided some insight into the role that mutant GCAP1 may play in retinal pathology. The murine *Guca1a* and *Guca1b* genes, encoding GCAP1 and 2 respectively, are in a tail-to-tail array on chromosome 17, and this provided a simple strategy via a double knock-out mutation, to study the impact of a complete loss of GCAP function in the mouse [17]. Although the human disease associated with mutations in *GUCA1A* is attributed to gain-of-function mutations and the knock-out mice have no discernible retinal degeneration, this study has advanced our understanding of the regulation of guanylate cyclase activity by GCAP proteins.

A transgenic mouse line with a mutant Y99C bovine GCAP1 cDNA transgene driven by a rhodopsin promoter has been shown to exhibit a severe early-onset retinal disease with dysregulation of Ca^2+^-dependent retGC1 activation [18]. Disease in this transgenic mouse line differs therefore from the human disorder since mutant GCAP1 will be present only in rod photoreceptors, whereas the human disease was originally described as a cone dystrophy. Perhaps not surprisingly therefore, there are significant differences in the pattern and rate of loss of rods and cones in the transgenic mouse [18] compared to the human disease, indicating that the disease pathology generated by the mutant transgene is significantly different from the human disorder. The transgenic mouse is not therefore a suitable animal model for assessing disease treatment of the human disease.

We have therefore chosen to generate a more accurate model of the human disease via a gene targeting approach, by introducing a point mutation into the endogenous promoter. The advantage of such an approach is that the control of gene expression remains under the endogenous promoter, allowing the consequences of the mutation to be studied without the significant complications of over-expression associated with multiple copies of a transgene and heterologous regulation. Such targeted introductions of mutations to generate “knock-in” mutant mice, have recently been used to create models of ocular disease that closely mimic their human counterparts [19]–[21]. In this study we have generated a knock-in model of the disease-causing E155G amino acid substitution in EF4 of GCAP1 [22]. Our model demonstrates that mutant GCAP1, when under normal expression control, causes both rod and cone photoreceptors to lose function and degenerate, with cone cells being more severely affected, in keeping with the human disease phenotype. This will enable a greater understanding of the progression and mechanisms of disease in COD3 patients and provide a more informative and reliable means of investigating treatment strategies.

## Results

### Gene targeting approach yields ‘knock-in’ mice with mutant *Guca1a*

**Figure 1:**
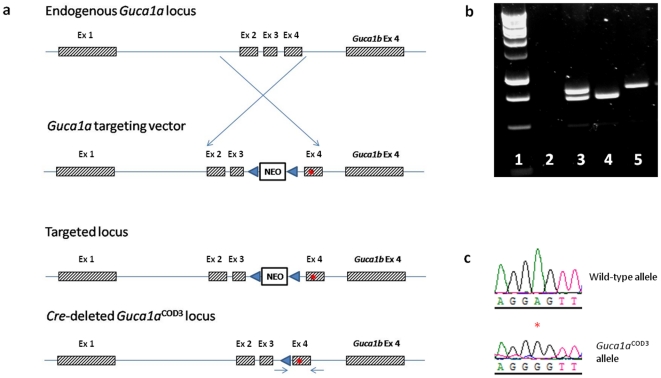
Generation of *Guca1a* ^COD3^ ‘knock-in’ mice with E155G mutation in GCAP1. (**a**) A vector targeting the endogenous *Guca1a* locus was constructed to include an A-to-G transversion at nucleotide position 19 in exon 4 of *Guca1a* (red circle), as well as a *loxP*-flanked (blue arrow heads) neomycin resistance gene within intron 3. Following *Cre*-mediated excision of the *Neo* selectable marker, the resulting locus contained the A-to-G change in exon 4 plus a residual 34 bp *loxP* sequence in intron 3. This was used to distinguish between mutant and native alleles by PCR – the position of the primers used is indicated by the small blue arrows on the *Cre*-deleted *Guca1a* locus. (**b**) PCR amplicons from wild-type, *Guca1a* ^+/COD3^ and *Guca1a* ^COD3/COD3^ mice (lanes 3, 4 and 5 respectively), with 1 kb DNA ladder (lane 1) and no-DNA control (lane 2). The wild type allele generates a band at 734 bp whereas the mutant allele generates a 768 bp band which includes the residual intronic *loxP* sequence. Wild-type mice have therefore a single band at 734 bp, homozygous *Guca1a* ^COD3/COD3^ mice have a single band at 768 bp, and heterozygous *Guca1a* ^+/COD3^ mice have both bands. (**c**) Sequence of wild type and targeted *Guca1a* allele showing A-to-G transversion at nucleotide position 19 of exon 4.

A plasmid containing the full murine *Guca1a* locus, with the introduction of an A-to-G substitution at base-pair 19 of exon 4 and a neomycin-resistance gene in intron 3, was generated as shown in **Figure 1** and used for the targeting of the *Guca1a* gene. The resulting *Guca1a* ^COD3^ mice were genotyped using primers that flanked a residual intronic *loxP* sequence to identify gene-targeted allele; the PCR products from homozygous and heterozygous mice (shown in **Figure 1b**) were sequenced to determine whether the *loxP* sequence segregated with the A-to-G transversion. As shown in **Figure 1c**, the point mutation was present in all targeted alleles. The targeted or mutant allele is referred to here as *Guca1a* ^COD3^. From the F1 mice, heterozygous *Guca1a* ^+/COD3^ and homozygous *Guca1a^COD3/^* ^COD3^ mutant mice were generated; in this study we present data from both mutant genotypes as the former represents a close correlate to patients with COD3, whilst the latter may provide further insights into the role that mutant GCAP1 plays in causing cone and cone-rod dystrophy.

### GCAP1 levels and effect on other photoreceptor proteins in pre-symptomatic mutant mice

**Figure 2:**
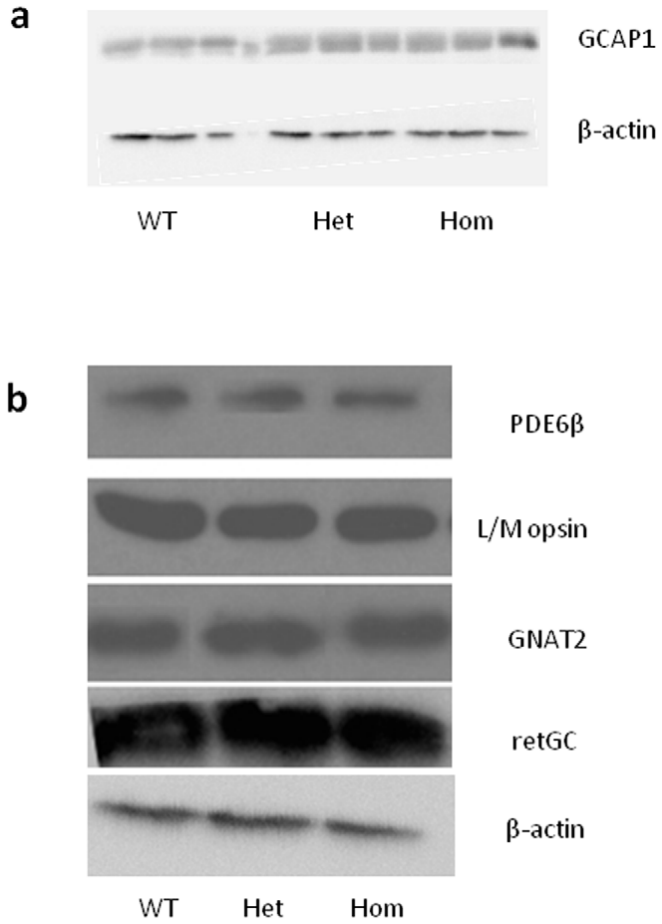
Photoreceptor-specific protein expression levels in young *Guca1a* ^COD3^ ‘knock-in’ mice. Western blots from retinal homogenates taken from six week-old wild-type (WT), heterozygous (Het) and homozygous (Hom) mutant mice. Equally loaded protein extracts were blotted with anti-β-actin as a loading control. (**a**) α-GCAP1 antibody DSC-1 with the retinae from three eyes per genotype. (**b**) PDE6β (rod-specific), L/M opsin, Gα_T2_ (GNAT2 - cone-specific α-transducin) and retGC (rod and cone) antibodies, with the retinae from a single eye per genotype. In all cases, there are no discernible differences in staining between genotypes.

The gene targeting approach enables us to study the consequences of a clinically relevant mutation in an endogenous gene, leaving the rest of the gene intact and under normal expression control. To ensure the introduction of the E155G mutation in GCAP1 did not alter the expression level of GCAP1 protein prior to the onset of retinal dysfunction, a time point of 6 weeks of age was selected when retinal structure and function appeared to be normal (see below). The amount of GCAP1 protein present was assayed by Western blotting; as shown in **Figure 2a**, there is no substantive change in retinal GCAP1 protein level in either heterozygous or homozygous mutant mice, compared with wild-type mice of similar age. This analysis was then extended to a panel of photoreceptor markers: rod-specific phosphodiesterase (PDE6β) cone-specific L/M opsin, cone-specific transducin (GNAT2), and retGC. Western blot analysis again showed no substantive changes in the protein levels of any of these photoreceptor-specific proteins in heterozygous or homozygous mice compared with wild-type littermates (**Figure 2b**). The levels of these phototransduction cascade proteins are unaffected therefore at 6 weeks of age by the production of mutant GCAP1, indicating that photoreceptors develop normally in mutant mice prior to the onset of the pathological changes, described below.

### Elevated intracellular cGMP levels in mutant mice

Since mutant GCAP1 protein has been shown *in vitro* to result in loss of Ca^2+^-sensitivity and the constitutive activation of retGC1 [22], mutant mice would be expected to show elevated levels of cGMP production. Therefore, cGMP levels were measured from the retinae of 6 week-old mutant mice and wild-type littermates. As shown in **Table 1**, cGMP levels in homozygous mice were significantly elevated compared with wild-type littermates, with heterozygous mice also showing elevated levels that fall just short of statistical significance. This indicates that prior to the development of an overt phenotype as measured by retinal function, cGMP levels are already elevated in mutant mice.

**Table 1:** cGMP levels in retinae of *Guca1a* ^COD3^ ‘knock-in’ mice.

Genotype	cGMP	P values
	fmol/µg protein	(mutant v wild type)
**Wild type**	77.0±9.3	
***Guca1a^+/^*** ^**COD3**^	100.6±23.1	0.09
***Guca1a^COD3/^*** ^**COD3**^	124.6±16.0	<0.01

cGMP levels were measured in six week-old mice, before the onset of degeneration as evidenced by retinal structure and function. Levels of cGMP were corrected for total protein content of each sample.

### Photoreceptor function in mutant mice

**Figure 3:**
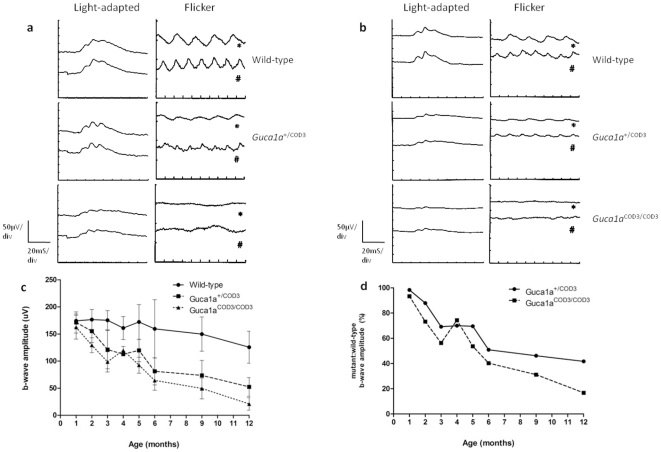
Electroretinography of *Guca1a* ^COD3^ ‘knock-in’ mice. (**a**) Representative ERG traces recorded at five months of age. Light-adapted, cone-mediated ERG (upper trace, right eye, lower trace, left eye) is reduced in *Guca1a* ^+/COD3^ and *Guca1a* ^COD3/COD3^ mice compared to wild-type littermates, as is the cone-mediated flicker response to 10- and 15-Hz stimuli (* and # respectively). (**b**) Representative ERG traces recorded at 12 months of age. There is a greater reduction in cone function in mutant mice, with light-adapted flash responses further attenuated and flicker responses almost extinguished. (**c**) Averaged photopic, cone-mediated b-wave amplitudes from wild-type, *Guca1a* ^+/COD3^ and *Guca1a* ^COD3/COD3^ mice recorded over a twelve month period from birth. There is a progressive loss of cone function over time in both heterozygous and homozygous mutant mice compared to wild-type littermates (*n = 8* per genotype). (**d**) ERG b-wave amplitudes from *Guca1a* ^+/COD3^ and *Guca1a* ^COD3/COD3^ mice plotted as a percentage of wild-type amplitudes over a twelve month period from birth.

**Figure 4:**
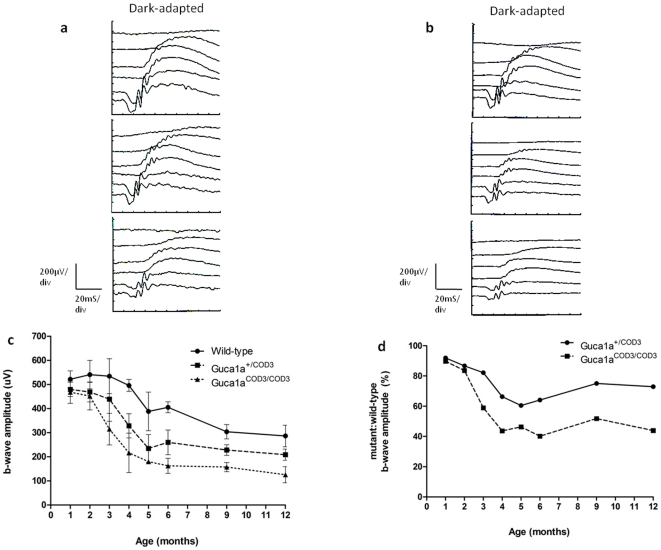
Reduced rod function in *Guca1a* ^COD3^ ‘knock-in’ mice. (**a**) Representative ERG traces taken from dark-adapted 5 month-old wild-type, *Guca1a* ^+/COD3^ and *Guca1a* ^COD3/COD3^ mice, showing a reduction in a- and b-wave amplitude in mutant mice in the rod-dominated ERG in response to a series of increasing stimulus intensities. (**b**) Representative ERG traces taken from dark-adapted 12 month-old wild-type, *Guca1a* ^+/COD3^ and *Guca1a* ^COD3/COD3^ mice, showing that rod function is relatively spared when compared with cone function. (**c**) Averaged scotopic, rod-dominated b-wave amplitudes from wild-type, *Guca1a* ^+/COD3^ and *Guca1a* ^COD3/COD3^ mice recorded over a twelve month period from birth. Mean values were calculated from b-wave amplitudes from 100 mcds/m^2^ flash intensity, which corresponds to a rod-only response. Note the age-related decline in wild-type mice. (**d**) Rod b-wave amplitudes in *Guca1a* ^+/COD3^ and *Guca1a* ^COD3/COD3^ mice plotted as a percentage of wild-type amplitudes over a twelve month period from birth.

ERGs were recorded from mutant mice and wild-type littermates to determine the effects of the E115G mutation in GCAP1 on retinal function. Although retinal function in young mutant mice appeared to be normal, from three months of age cone function in heterozygous *Guca1a^+/^* ^COD3^ mice was significantly reduced. At five months of age, there was a marked reduction in the cone-mediated light-adapted (photopic) ERG in mutant mice (**Figures 3a, c**), with a b-wave amplitude of 119.7±20.5 µV in *Guca1a^+/^* ^COD3^ mice compared with 172.2±32.0 µV in wild-type littermates (p = 0.003). The 10 Hz and 15 Hz cone flicker ERG responses were severely depressed in *Guca1a^+/^* ^COD3^ mice, in keeping with profound loss of cone function (**Figure 4a**). This reduction in cone responses progressed with age, with 12 month-old *Guca1a^+/^* ^COD3^ mice retaining 42% of the photopic ERG b-wave amplitude of their wild-type littermates (**Figure 3a, c**). Homozygous *Guca1a^COD3/^* ^COD3^ mice also showed a significant reduction in cone function, with light-adapted b-waves down to 92.4±14.7 µV and flicker responses absent at five months of age (**Figure 3a**). As a proportion of age-matched wild-type littermates, 12 month-old *Guca1a^COD3/^* ^COD3^ mice retain just 17% cone function (**Figure 3b, d**).

There was also a reduction in the rod-mediated dark-adapted (scotopic) ERG that was apparent from three months of age. At five months of age (**Figure 4a**), dark-adapted b-wave amplitudes from *Guca1a^+/^* ^COD3^ mice (234.6±57.3 µV) were significantly reduced compared with those from wild-type littermates (388.2±80.1 µV, p<0.001). The loss of rod function in mutant mice continued over time; however, there was also a concomitant reduction in rod function in wild-type littermates such that *Guca1a* ^+/COD3^ mice retained 74% of wild-type scotopic b-wave amplitudes (**Figure 4c, d**). It is noteworthy therefore that overall, cone photoreceptor dysfunction was more severe than rod dysfunction in *Guca1a* ^+/COD3^ mice. Homozygous *Guca1a^COD3/^* ^COD3^ mice suffered a greater loss in rod function (average scotopic b-wave amplitude was 179.7±3.6 µV at five months of age); at 12 months of age homozygous *Guca1a^COD3/^* ^COD3^ mice retained only 44% of the b-wave amplitude of wild-type littermates.

### Impaired rod recovery kinetics in mutant mice as measured by paired-flash ERG

**Figure 5:**
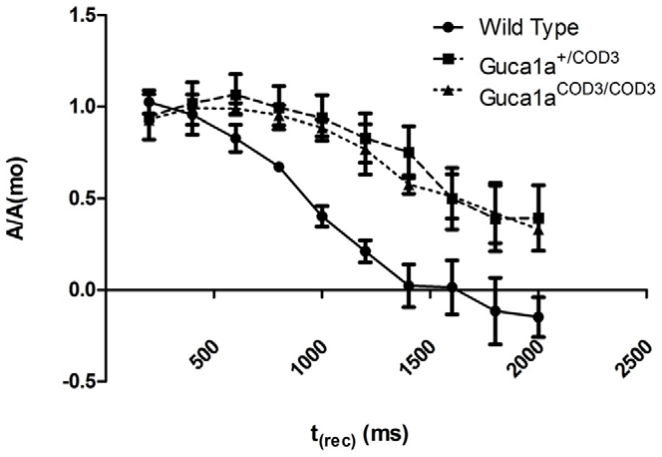
Impaired recovery from bright flash of *Guca1a* ^COD3^ ‘knock-in’ mice. **T**he a-wave amplitude from a bright test flash, presented at varying times after a conditioning flash, is plotted as a proportion of the amplitude elicited by the conditioning flash. A value of 0 indicates the amplitude from the second flash is the same as that from the first; full recovery from a bright conditioning flash occurs within two seconds in wild-type mice, and the second flash elicits an amplitude half that of the first when the inter-stimulus interval is approximately 1000 ms. This recovery time is extended to 1600 ms in both heterozygous and homozygous knock-in mice, whilst the amplitude from the second flash fails to reach that elicited by the first flash during the time period of recording.

Since GCAP1 has a role in recovery following activation of the phototransduction cascade, we used a paired-flash ERG method to determine whether the rate of recovery from a bright flash was disturbed in mutant mice. Paired flash responses have been used successfully to determine the rate of recovery of photoreceptor currents *in vivo*, [23]–[30], and are known to be reduced in patients with COD3 [15]. Paired-flash ERG responses were therefore used to monitor the kinetics of recovery in dark-adapted mutant mice and wild-type littermates. Since <5% of the saturated a-wave is due to cones [31]–[33], the a-wave in these responses can be attributed almost entirely to rod function. Dark-adapted mice were exposed to a bright conditioning flash, followed by a second probe flash at varying intervals. The a-wave amplitudes elicited by the latter were then plotted as a proportion of the former against time (**Figure 5**). In wild-type mice, the a-wave from the probe flash recovers fully within two seconds, whereas in both *Guca1a^+/^* ^COD3^ and *Guca1a^COD3/^* ^COD3^ mice, recovery was delayed, with only around 65% recovery of the a-wave within 2 seconds of the conditioning flash, with the time to half-recovery (where the probe flash gives rise to an a-wave amplitude half that of the first) extended from 1000 ms in wild type to 1600 ms in heterozygous and homozygous mutant mice. These observations clearly demonstrate that, *in vivo*, there is impaired recovery of rod photoreceptors from a bleaching flash in mutant mice.

### Cone and rod opsin expression and cone loss

**Figure 6:**
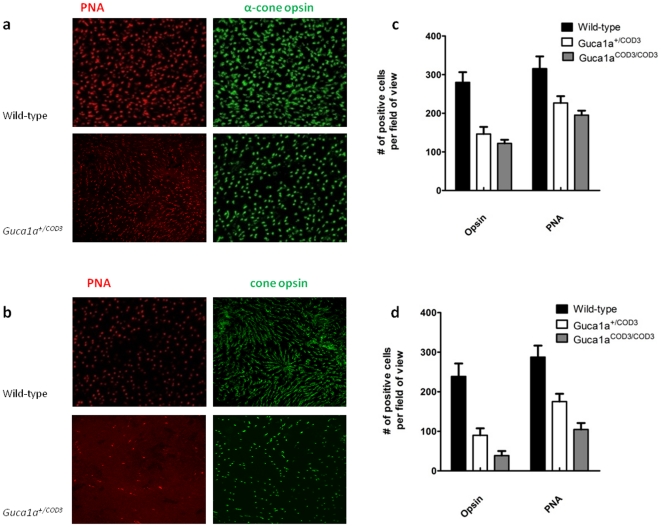
Mutant GCAP1 leads to progressive loss of cone photoreceptors. (**a**) Representative retinal flat mounts taken from 5 month-old mice stained with PNA and anti-cone opsin antibody. There are fewer PNA-positive cone cells present in mutant compared to wild-type retinae, as well as a fewer opsin-expressing cells in mutant versus wild-type retinae. Quantitative analysis shows that there are significantly fewer cone opsin-expressing cells in mutant retinae than PNA-positive cells, although the labelling of both markers is significantly reduced in *Guca1a* ^+/COD3^ and *Guca1a* ^COD3/COD3^ mice as compared to wild-type littermates. (**b**) Representative retinal flatmounts taken from 12 month-old mice confirms progression of cone degeneration; in mutant mice, there are fewer opsin-positive cells at 12 months compared to wild-type littermates.

**Figure 7:**
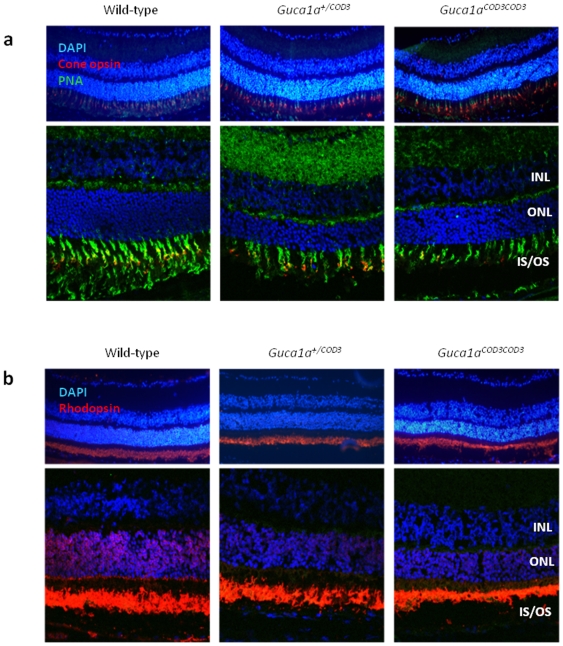
Photopigment expression in ‘*Guca1a* ^COD3^ ‘knock-in’ mice. Representative cryosections from five month-old mice stained with DAPI and (**a**) a combined anti-L/M- and S-cone opsin antibody (red) and PNA (green), and (**b**) an anti-rhodopsin (rod opsin) antibody (red). For both, upper panels show fluorescent light micrographs, and lower panels show single slices from confocal microscopy. The expression of both cone and rod opsin appears similar in both *Guca1a* ^+/COD3^ and *Guca1a* ^COD3/COD3^ mice compared with wild-type littermates, although the rod opsin staining indicates a shortening of rod outer segments. PNA staining (green) in (**a**) indicates a reduced number of cones in the mutant retinae which is more pronounced in homozygous than in heterozygous mutant mice. INL inner nuclear layer, ONL outer nuclear layer, IS/OS inner segment/outer segment.

To further assess the effect of mutant GCAP1 on other components of the phototransduction cascade, the expression of L/M- and S-opsin in cones and rod opsin (rhodopsin) in rods was examined immunohistochemically using specific antibodies on frozen retinal tissue. At five months of age, there were fewer cone photoreceptors expressing cone opsins in *Guca1a^+/^* ^COD3^ and *Guca1a^COD3/^* ^COD3^ mice (**Figure 6a**). In contrast, the expression of rod opsin in rod outer segments shows less of a decline (**Figure 6b**). Flat mounts were then used to quantify the number of opsin-expressing cones remaining in mutant retinae at five and 12 months of age. At five months of age there was a significant loss of cones; heterozygous *Guca1a^+/^* ^COD3^ and homozygous *Guca1a^COD3/^* ^COD3^ mice retained 52% and 44% of opsin-expressing cells compared with wild-type littermates (**Figure 7a and c**). In keeping with these findings, there was also a loss of PNA-positive cells; at five months of age *Guca1a^+/^* ^COD3^ mice retained 72% and *Guca1a^COD3/^* ^COD3^ mice retained 62% PNA-positive cells when compared with wild-type littermates. The loss of cone cells was progressive, and at twelve months of age heterozygous *Guca1a^+/^* ^COD3^ mice retained 38% and homozygous *Guca1a^COD3/^* ^COD3^ mice retained only 16% of cone opsin-positive cells compared with age-matched wild-type littermates (**Figure 7a and c**). The loss of PNA-positive cells also continued over time, such that at twelve months of age *Guca1a^+/^* ^COD3^ mice retained 61% and *Guca1a^COD3/^* ^COD3^ mice retained 36% as compared with wild-type littermates.

### Photoreceptor loss in mutant mice

**Figure 8:**
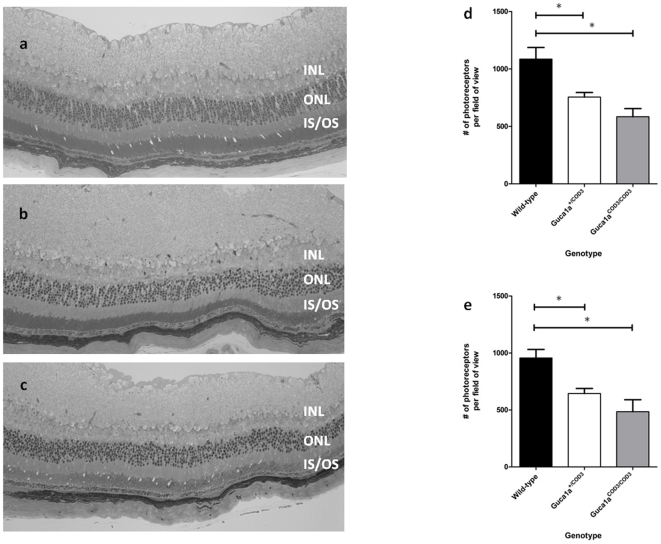
E155G mutation in GCAP1 causes photoreceptor degeneration. Photomicrographs of resin-embedded sections from (**a**) wild-type (**b**) *Guca1a* ^+/COD3^, and (**c**) *Guca1a* ^COD3/COD3^ mice at five months of age. There is a marked thinning of the outer nuclear layer (ONL) in mutant eyes as shown by the loss of photoreceptor nuclei. There is also an apparent shortening of outer segment length, particularly in the *Guca1a* ^COD3/COD3^ homozygous mice. Photoreceptor loss in *Guca1a* ^+/COD3^ and *Guca1a* ^COD3/COD3^ mice was quantified by counting photoreceptor nuclei in photomicrographs of sections from paraffin-embedded eyes taken at fixed positions around the optic nerve. At five months of age (**d**), there is 30% loss in *Guca1a* ^+/COD3^ and a 46% loss in *Guca1a* ^COD3/COD3^ mice. At 12 months of age (**e**), this has progressed to a 32% loss in *Guca1a* ^+/COD3^ and a 49% loss in *Guca1a* ^COD3/COD3^ mice. * indicates statistical significance at the 1% probability level. Note that in the lower panels of (a), PNA shows non-cone staining above the ONL and INL. INL inner nuclear layer, ONL outer nuclear layer, IS/OS inner segment/outer segment.

Since GCAP1 is expressed in both rod and cone photoreceptors, with evidence of greater expression in cones than rods [34], and mutant mice show a significant reduction in rod function, we performed histological analysis of the retina at various ages to ascertain whether the loss of retinal function with age was associated with disturbed retinal architecture. The retina appeared to develop normally, with no evident loss of photoreceptors at two months of age, in both *Guca1a^+/^* ^COD3^ and *Guca1a^COD3/^* ^COD3^ mice (data not shown). However, at five months of age there was a marked reduction in the thickness of the outer nuclear layer (ONL) in both heterozygous and homozygous mutant mice, indicating a loss of photoreceptor nuclei. As evident in resin-embedded semi-thin sections, the remaining photoreceptor cells appear to have shortened and/or dysmorphic outer segments, both of which are common features of retinal degeneration [35]–[39] (**Figure 8a, b** and **c**). Quantitative analysis performed on paraffin-embedded sections showed that at five months of age, the loss of photoreceptors in mutant mice was significant, with *Guca1a^+/^* ^COD3^ mice retaining 69% of cells in the ONL compared with wild-type littermates (**Figure 8d**). However, the rate of loss of photoreceptor nuclei appears to decline with age, since by 12 months of age *Guca1a^+/^* ^COD3^ mice still retained 67% of cells in the ONL compared with wild-type littermates (**Figure 8e**). *Guca1a* ^COD3/COD3^ mice also showed a thinning of the ONL, retaining 54% of cells in the ONL compared to wild-type littermates at five months of age (**Figure 8d**) and 51% at 12 months (**Figure 8e**).

## Discussion

A key step in phototransduction in vertebrates is the closure of cGMP-gated cation channels and the continued active efflux of Ca^2+^ as a result of a cascade initiated by photon capture by the visual pigment, with subsequent breakdown of cGMP by the activation of phosphodiesterase activity. This process is reversed by the synthesis of cGMP at low intracellular Ca^2+^ concentrations via the activation of guanylate cyclase by GCAPs. In the mouse model characterised in this study, the regulation of this latter process has been altered by the introduction of a single nucleotide missense mutation in the endogenous *Guca1a* gene using gene targeting. The mutated gene encodes a E155G substitution in EF4 of the GCAP1 protein; Ca^2+^ binding to the mutant GCAP1 is reduced to only two hands (EF2 and EF3) and thereby reduces the feedback loop [17], [40] whereby cyclase activity is reduced as Ca^2+^ concentrations in photoreceptors are brought back to dark-state levels. Consistent with this, we have shown that retinal levels of cGMP in mutant mice are elevated prior to the development of any overt pathology.

The retinal disease seen in human patients with dominant mutations in *GUCA1A* was originally described as an isolated cone dystrophy, but recent evidence suggests that secondary loss of rod function may occur in some patients, particularly at later stages of disease [16]. The mouse mutant confirms the involvement of cones and rods, with both showing a progressive decline in function from 3 months of age as determined by ERG responses; although, in keeping with the human disorder, the decline in cone-mediated responses was greater than the decline in rod-mediated responses once the age-related loss of rod function is taken into account. Prior to the 3 month time point, ERGs recorded in wild type and mutant mice were indistinguishable, as was retinal morphology and the expression of cone and rod photoreceptor markers, indicating that retinal function and structure was initially normal. As the disease developed in *Guca1a* ^COD3^ mutant mice, there was a progressive reduction in the thickness of the photoreceptor cell layer, a progressive depression in ERG amplitude and a reduction in the number of cones. Although a previous study describing a transgenic mouse carrying a Y99C mutant bovine GCAP1 transgene also showed significant rod degeneration, this can be attributed to the fact that the transgene was expressed predominantly – if not exclusively – in rods [16]. In direct contrast, the phenotype in the model characterised here, with a greater impact on cones than on rods, is likely to be a direct consequence of the point mutation in GCAP1.

A role for GCAP1 in phototransduction in both rods and cones is indicated by various studies of GCAP knock-out mice. Mice with a double GCAP1 and GCAP2 knock-out show an altered response of rods to saturating flashes of light which is not rescued by the production of GCAP2 from a transgene [17], whereas the degree of recovery post-flash in rods [31] and cones [41] has been shown to correlate with the level of GCAP1 expression in these mice when expressing a GCAP1 transgene. GCAP2 is also capable of regulating cGMP production by retGC1 in a Ca^2+^-dependent manner [17]. Since GCAP2 is predominantly expressed in rods [4], [42], [43], the loss of Ca^2+^-sensitivity due to the E155G mutation in GCAP1 may be compensated for by GCAP2 to a greater extent in rods than in cones, and may thereby account for the increased loss of cones compared with rods in both the animal model and human disease. In contrast, as shown by the GCAP1 and GCAP2 double knock-out, the loss of all GCAP function does not result in retinal degeneration [17].

The causal relationship between photoreceptor degeneration and mutant GCAP1 has yet to be fully established. Previous work with transgenic mice expressing mutant GCAP1 protein has shown elevated levels of intracellular Ca^2+^ [18]. This is also the predicted consequence of the elevated cGMP levels seen in the *Guca1a* ^COD3^ mutant mice. Elevated levels of Ca^2+^ have been shown to activate apoptotic pathways in rod photoreceptors [44] and may therefore be the major factor in the retinal degeneration in these mice, and in the human disease. The same may be the case in *rd1* mutant mice which either lack [45] or have severely reduced levels [46] of the cGMP-phosphodiesterase. It has also been reported in one study that D-*cis*-diltiazem, a calcium-channel blocker, rescues photoreceptors and preserves visual function in these mice [47] although other studies have failed to confirm these findings [48], [49].

Both heterozygous and homozygous *Guca1a* ^COD3^ mutant mice showed a significant delay in the recovery of the rod ERG a-wave after a bright conditioning flash. *In vitro*, mutant E155G GCAP1 results in a reduced sensitivity of cyclase activity to Ca^2+^ inhibition [22], and the elevated levels of cGMP seen in the retinae of the *Guca1a* ^COD3^ mutant mice indicate that the mutant GCAP1 is having a similar effect *in vivo*, so the delay in recovery is presumably a consequence of these elevated levels of cGMP. A delay in recovery of the rod a-wave is also seen in mice lacking both GCAP1 and GCAP2. This delay was reversed by the expression of GCAP1 via a transgene in a dose-dependent manner [31], and the same was found for the delay in the cone response [41]. Therefore, in both the GCAP1 knock-out and the E155G GCAP1 knock-in mice, cyclase activity remains elevated in the absence of GCAP-mediated Ca^2+^ regulation. Importantly, this delay in rod recovery is also a salient feature of the human disease, as described in the case of an N104K mutation in GCAP1 [15].

Since the phenotype presented here can be attributed to a single point mutation introduced into the native gene and independent therefore of positional effects and copy number variations resulting from transgenic lines generated by pronuclear injections of DNA constructs, we believe that the *Guca1a* ^COD3^ mutant mouse line represents a more accurate model of human cone dystrophy 3, and displays all the characteristic phenotypic hallmarks of the human disorder. In addition, the mouse model has demonstrated that cGMP levels are elevated prior to any depression in retinal function, indicating that this may be the trigger for the subsequent degenerative changes, and that there is a significant loss of photoreceptors as the disease progresses, although this is less evident for rods than for cones. The knock-in mouse model is likely to prove therefore to be a very useful platform for the testing of potential treatments such as pharmacological intervention and viral vector-mediated genetic therapies.

## Materials and Methods

### Ethics statement

All animal studies were carried out under the Animals (Scientific Procedures) Act 1986 under a project license PPL70/6115 issued by the UK Government Home Office and conducted in accordance with protocols approved by the Animal Ethics Committee of the UCL Institute of Ophthalmology. All efforts were made to minimize the number and suffering of animals used in these experiments.

### Generation of *Guca1a* ^COD3^ ‘knock-in’ mice

A plasmid construct targeting the endogenous mouse *Guca1a* locus and including an A-to-G transversion at codon 155 was transfected into 129 Sv mouse embryonic stem cells and resultant neomycin-resistant clones were injected into blastocysts for the production of chimeric mice. The neomycin cassette was flanked by *loxP* sequences to allow for its subsequent excision by crossing chimeric mice with mice expressing the *Cre* recombinase under the control of a ubiquitous CMV promoter. The screening of the resultant mice by PCR-amplification and sequencing of the region confirmed the presence of the point mutation and the successful removal of the neomycin cassette in the *Cre*-deleted mutant GCAP1 allele. These F1 mice were genotyped using PCR primers that flanked the targeted exon and the residual *loxP* sequence; the targeted allele, which included the single surviving intronic *loxP* sequence, gave a larger amplification product than the wild-type allele (**Figure 1**). The resultant PCR products were directly sequenced to verify that the A-to-G transversion segregated with the targeted allele.

### Western blotting

Six week-old *Guca1a* ^COD3^ mice and wild-type litter-mates were sacrificed by cervical dislocation and the eyes were removed. Retinae were dissected away from lens and RPE/choroid, and for analysis of cytoplasmic proteins (i.e. GCAP1, β-actin, PDE6β) were placed in RIPA buffer with added protease inhibitor cocktail (Sigma Aldrich, Gillingham, UK). Cell membranes were disrupted using a sonicator with a micro-tip (Soniprep 150, MSE London UK). Lysates were archived at −80°C. For analysis of membrane-bound proteins (retGC, GNAT2, cone opsins) membrane fractions were prepared by homogenising tissue in low salt homogenization buffer (10 mM MOPS pH 7.3, 5 mM mercaptoethanol, 20 µg/ml leupeptin, 1 mM PMSF) and the cells left to swell on ice for 10 min. The cells were lysed by five passages through a 0.5 mm gauge syringe needle. The salt content of the homogenate was then raised to 0.25 M with 5 M NaCl and the homogenate was spun at 4°C for 5 min at 900 *g* to remove large debris. Cell membranes were pelleted by further centrifugation of the supernatant at 14 926 *g* in a pre-cooled 4°C centrifuge for 30 min. Membrane pellets were resuspended in 150 µl of low salt homogenization buffer. Equal amounts of protein, determined by a Lowry-based colorimetric protein assay performed in triplicate (DC protein assay kit, Bio-Rad, Hemel Hempstead UK) compared to a bovine serum albumin standard curve, were run on a 9% (w/v) reducing SDS polyacrylamide electrophoresis gel. After semi-dry transfer to PVDF membrane (Millipore Watford UK) and blocking in 5% (w/v) non fat milk, 1% (w/v) bovine serum albumin in PBS-T (phosphate buffered saline with 0.005% Tween-20), membranes were incubated with primary antibodies. After washing in PBS-T, secondary antibody was incubated at a concentration of 1∶5000–1∶10 000 for 1 hour at room temperature (Pierce Immunopure goat anti-rabbit and anti-mouse IgG (H+L) HRP conjugated, Perbio Science UK Ltd., Northumberland UK). Chemiluminescence detection was performed using a Fujifilm LAS-1000 Luminescence Image Analyser after incubation with enhanced luminescence reagent (ECL plus GE Healthcare UK Ltd. Amersham, UK).

### Measurement of intracellular cGMP levels

Mice were sacrificed in the dark under infra-red illumination and retinae were dissected away from lens and RPE/choroid. Manufacturer's instructions for a cGMP competition ELISA were followed to assay cGMP levels (cGMP BIOTRAK assay, GE Healthcare UK Ltd. Amersham, UK). Briefly, cGMP was extracted from the retina by homogenisation in 200 µL ice-cold 6% (w/v) tricholoroacetate, followed by centrifugation for 30 minutes at 2,000 *g*. The cell pellet obtained from this cGMP isolation step was homogenised in RIPA buffer with added protease inhibitor cocktail (Sigma Aldrich, Gillingham, UK), and the amount of total protein in the sample quantified using a Lowry-based colorimetric protein assay performed in triplicate (DC protein assay kit, Bio-Rad, Hemel Hempstead UK) compared to a bovine serum albumin standard curve. The total protein content of each sample was used to correct the final cGMP value obtained per µg of protein. The supernatant containing cGMP was then washed four times with water-saturated diethyl ether (Sigma Aldrich Ltd., Gillingham, UK), with the aqueous phase recovered after each wash. After final wash, the sample was placed in a vacuum concentrator to allow evaporation of solvent and recovery of cGMP pellet which was resuspended in 200 µL 1× assay buffer. Samples were then applied in triplicate to a 96-well plate that was pre-coated with anti-cGMP antibody, together with a standard curve of cGMP at between 50 and 128,000 fmol. 100 µL of a separate anti-cGMP antibody was then applied and incubated at 4°C overnight, followed by 50 µL horseradish peroxidase-conjugated cGMP; after incubation at 4°C for four hours, the plate was washed and TMB substrate applied to all wells. The coloured product from the hydrolysis of this substrate was then quantified using an automated plate reader at 630 nm, with cGMP in the retinal samples competing out the cGMP in the assay kit, thereby resulting in a lower optical density reading.

### ERG recordings

All animals were dark-adapted overnight (16 h), anaesthetized and prepared for ERG recording as previously described [50]. Mice were then placed on a heated platform and ERGs recorded using commercially available equipment (Espion E2; Diagnosys LLC, Massachusetts USA) after corneal contact electrodes and midline subdermal reference and ground electrodes were placed. Bandpass filter cut-off frequencies were 1 and 300 Hz. Photopic single-flash recordings were obtained at light intensities increasing from 0.1 to 10000 mcds/m^2^ presented in a ganzfeld dome. Ten responses per intensity level were averaged with an interstimulus interval of 10 s (0.1, 1, 10 and 100 mcds/m^2^) or five responses per intensity with a 60 s interval (3000, 5000 and 10000 mcds/m^2^). Light-adapted, cone flicker responses were elicited in the presence of a 41 cd/m^2^ rod-desensitizing white background with the flashes of 10000 mcds/m^2^ presented at frequencies of 10 and 15 Hz. Dark-adapted, rod dominated responses were elicited in the dark using white light of intensities ranging from 0.001 to 3000 mcds/m^2^, with 10 responses per intensity and an interstimulus interval of 5 s (0.1, 1, 10 and 100 mcds/m^2^) or five responses per intensity with a 60 s interval (1000 and 3000 mcds/m^2^).

Paired-flash ERGs were recorded by saturating the rods with a 25000 mcds/m^2^ flash at time T_0_, followed *t*-milliseconds later by a test flash of equal intensity. The ratio between the a-wave elicited by the test flash (A) and that from the probe flash (A_mo_) was then calculated, and the Log_10_ of this value was plotted as a function of time *t* as a measure of recovery which approached one as time *t* was increased and the response to the test flash approached that to the probe flash.

### Immunohistochemistry

Animals were terminally anaesthetised and eyes were fixed by intracardiac perfusion with 1% paraformaldehyde in PBS pH 7.4 and post fixed in 1–4% paraformaldehyde at room temperature. Retinal cryosections were incubated for 1 h at room temperature in PBS with 1% bovine serum albumin (BSA, Sigma Aldrich Ltd. Gillingham, UK) and 5% normal goat serum (Abd serotec, Kidlington UK). For retinal flatmounts, 0.05% (v/v) Triton X-100 (Sigma Aldrich Ltd., Gillingham UK) was added to the blocking solution. For retinal cryosection analysis (n = 4 eyes for each genotype), 12 µm retinal sections were incubated with 4D2 anti-rhodopsin antibody or anti-cone opsin antibodies at 1∶250 dilution in blocking solution for 1 hour at room temperature. For retinal flatmount examination (n = 4 eyes for each genotype), eyecups with the cornea and lens removed were incubated with anti-cone opsin antibodies at a dilution of 1∶100 in blocking solution for 4 hours at 37°C. Counterstaining with biotinylated PNA (Sigma Aldrich, Gillingham, UK) was performed at a concentration of 0.1 mg/ml in PBS overnight at 4°C. Retinas were washed extensively in PBS and dissected from the eyecup, radial cuts were made to flatten the retina, and the retinas were mounted with fluorescent aqueous mounting medium (Dako Ltd., Ely UK), photoreceptor cell layer uppermost, using a coverslip.

### Semi-thin sections

Prior to fixation, eyes for semithin sectioning were orientated with a suture on the nasal aspect of the eyes. Eyes were immersion fixed in 3% glutaraldehyde and 1% paraformaldehyde buffered to pH 7.4 with 0.07 M sodium cacodylate-HCL. After a minimum of 12 h of fixation, the cornea and lens were removed. The posterior segments were then osmicated for 2 h with a 1% aqueous solution of osmium tetroxide and dehydrated through ascending alcohols (50–100%, 10 min per step). After three changes of 100% ethanol, the specimens were passed through propylene oxide (2×20 min) and left overnight in a 50∶50 mixture of propylene oxide and araldite. Following a single change to fresh araldite (5 h with rotation), the specimens were embedded and cured for 24 h at 60°C. Using the nasal suture, the eyes were embedded sagittally so that sectioning occurred in the vertical plane and the retinal sections contained the superior and inferior retina. Semithin (0.7 µm) sections were cut using a Leica Ultracut S microtome fitted with an appropriate diamond knife (Diatome histoknife Jumbo or Diatome Ultrathin). Semithin sections were stained with 1% toluidine blue and evaluated using a Leitz Diaplan microscope fitted with a Leica digital camera DC 500 for image capture.

### Paraffin histology and photoreceptor quantification

Following cervical dislocation, eyes were fixed for 24 h at 4°C by incubation in ice-cold neutral buffered 10% (v/v) formalin; a corneal puncture was created to allow the fixative to penetrate the eye. Eyes (n = 6 eyes for each genotype) were processed in an automated machine (Leica TP1020) then embedded in paraffin blocks for sectioning. Sections were cut on a microtome at 6 microns, and mounted on polylysine coated slides (Thermofisher Loughborough, UK). Sections were counterstained with haematoxylin (staining nuclei), and mounted using DPX (VWR, Leicester UK). Light microscope images were taken of sections at fixed positions in the eye in relation to the optic nerve head, and the number of photoreceptor nuclei in the ONL was counted in ten sections per eye.
